# A risk score for predicting atrial fibrillation in individuals with preclinical diastolic dysfunction: a retrospective study in a single large urban center in the United States

**DOI:** 10.1186/s12872-019-1024-4

**Published:** 2019-02-27

**Authors:** Dan L. Li, Renato Quispe, Nidhi Madan, Lili Zhang, Cynthia C. Taub

**Affiliations:** 1 Department of Medicine, Albert Einstein College of Medicine, Jacobi Medical Center, Bronx, NY USA; 2 Johns Hopkins Ciccarone Center for the Prevention of Heart Diseases, Baltimore, USA; 3 Department of Cardiology, Montefiore Medical Center, Albert Einstein College of Medicine, Jack D. Weiler Hospital, 1825 Eastchester Rd, Bronx, NY 10461 USA

**Keywords:** Atrial fibrillation, Preclinical diastolic dysfunction, Grade 1 diastolic dysfunction, Risk score

## Abstract

**Background:**

Left ventricular diastolic dysfunction has been shown to associate with increased risk of atrial fibrillation (AF). We aimed to examine the predictors of AF in individuals with preclinical diastolic dysfunction (PDD) - diastolic dysfunction without clinical heart failure – and develop a risk score in this population.

**Methods:**

Patients underwent echocardiogram from December 2009 to December 2015 showing left ventricular ejection fraction (LVEF) ≥ 50% and grade 1 diastolic dysfunction, without clinical heart failure, valvular heart disease or AF were included. Outcome was defined as new onset AF. Cumulative probabilities were estimated and multivariable adjusted competing-risks regression analysis was performed to examine predictors of incident AF. A predictive score model was constructed.

**Results:**

A total of 9591 PDD patients (mean age 66, 41% men) of racial/ethnical diversity were included in the study. During a median follow-up of 54 months, 455 (4.7%) patients developed AF. Independent predictors of AF included advanced age, male sex, race, hypertension, diabetes, and peripheral artery disease. A risk score including these factors showed a Wolber’s concordance index of 0.65 (0.63–0.68, *p* <  0.001), suggesting a good discrimination.

**Conclusions:**

Our study revealed a set of predictors of AF in PDD patients. A simple risk score predicting AF in PDD was developed and internally validated. The scoring system could help clinical risk stratification, which may lead to prevention and early treatment strategies.

**Electronic supplementary material:**

The online version of this article (10.1186/s12872-019-1024-4) contains supplementary material, which is available to authorized users.

## Background

Atrial fibrillation (AF) is the most common sustained cardiac arrhythmia in the general population [1], and is associated with increased risk of stroke, cardiovascular events, heart failure and mortality [2], profoundly impacting on both individual’s quality of life and health care burden [2, 3].

Preclinical diastolic dysfunction (PDD), defined as isolated diastolic dysfunction without clinical presentations of heart failure [4], is a common phenomenon in general population, with a prevalence of 27–36% [5–7]. Despite the absence of symptoms, PDD is characterized by pathological cardiac remodeling, and has been shown to associate with a significantly increased risk of heart failure and increased mortality [8, 9]. Left ventricular diastolic dysfunction has been shown to associate with increased risk of new-onset AF [10–14], as well as early AF recurrence post-ablation [15]. However, to the best of our knowledge, no studies have identified predicting factors of incident AF in PDD population. Identifying predictors of incident AF and high-risk individuals could help direct early preventive measures in PDD population, and therefore, potentially reduce mortality and morbidity.

In the present study, we aimed to: 1) investigate the predicting factors of new-onset AF in our multiracial cohort of patients with PDD, and 2) construct a risk score that predicts incidence of AF using easily available clinical variables.

## Methods

### Study population

We retrospectively included adult patients (age ≥ 18 years) who underwent echocardiogram in Montefiore Medical Center, Bronx, New York between December 21, 2009 and December 31, 2015 showing left ventricular ejection fraction (LVEF) of or above 50% and grade 1 diastolic dysfunction. Index echocardiogram was the first one performed during that time period if multiple echocardiograms were obtained. Patients with clinical diagnosis of heart failure, valvular heart disease or prevalent AF (from ICD codes) at the time of index echocardiogram were excluded from the study. We chose to select patients only with grade 1 diastolic dysfunction to represent PDD. The study protocol was approved by the Institutional Review Board of Albert Einstein College of Medicine.

### Baseline characteristics

Clinical data was collected from electronic medical record using Clinical Looking Glass™ - a patented software that collates medical records for research purposes.

Basic demographic characteristics included age, sex and self-reported race/ethnicity, in addition to socioeconomic score (SES), which was assessed using a summary score of wealth and income, and shown as a Z-score to reflect the deviation above (positive) or below (negative) the mean socioeconomic score of the New York state population. We used ICD codes to obtain information on prevalent comorbidities at the index time including hypertension (ICD 9 code 401 and ICD 10 code I10), diabetes (ICD 9 code 250 and ICD 10 codes E08-E13), myocardial infarction (ICD 9 code 410 and ICD 10 code I21), cerebrovascular disease (ICD 9 code 437.9 and ICD 10 codes I60-I69), peripheral artery disease (ICD 9 code 443.9 and ICD 10 code I73.9), chronic obstructive pulmonary disease (ICD 9 code 496 and ICD 10 code J44.9), chronic kidney disease (ICD 9 code 585.3 and ICD 10 code N18), and any malignancy (ICD 9 codes 140–208, and ICD 10 codes C00-C97) were defined by ICD-9 and 10 codes. Alcohol use disorders (ICD 9 code 291, 303, 305; ICD 10 code F10) and tobacco use disorders/nicotine dependence (ICD 9 code 305.1; ICD 10 codes F17 and Z72) were also collected via clinical diagnosis by ICD-9 and 10 codes. Laboratory values (serum levels of sodium, hemoglobin, creatinine), as well as use of medications (beta-blockers, angiotensinogen-converting enzyme inhibitors/angiotensin receptor blockers, calcium channel blocks, diuretics and statins) within 6 months before or at the time of inclusion were also gathered. The latest height and weight within 1 year of the index date were collected. Body mass index (BMI) was calculated as (weight in kg)/(height in m)^2^. Obesity was defined as BMI ≥ 30 kg/m^2^.

### Echocardiographic variables

Echocardiography was performed with Philips Sonos 7500 or IE-33 ultrasound systems (Philips Healthcare, Andover, MA). LVEF was calculated using either biplane Simpson’s method or Teichholz formula. Grade 1 diastolic dysfunction in the study was defined as mitral E/A ratio ≤ 0.8 and E/e’ ratio < 10 [16, 17], to represent impaired left ventricular relaxation without increased left ventricular filling pressure and/or increased pulmonary wedge pressure [18]. Other echocardiographic parameters including left atrial anteroposterior diameter (LAD, in parasternal long axis view), intraventricular septum thickness at end-diastole (IVSd), left ventricular posterior wall thickness at end-diastole (LVPWd), and left ventricular end-diastolic diameter (LVEDD) were collected. Left ventricular (LV) mass was calculated using formula LV mass (g) = 0.8 × 1.04 x [(IVSd (cm) + PWd (cm) + LVEDD (cm))^3^-LVEDD (cm)^3^] + 0.6. Body surface area (BSA) was estimated using the formula from Dubois and Dubois: BSA = 0.007184 x (height in cm)^0.725^ x (weight in kg)^0.425^ [19]. Left atrial diameter index (LAD index) was calculated as LAD adjusted by BSA.

### Outcome

The primary endpoint of the study was incident AF, defined as the first documented diagnosis of atrial fibrillation by ICD codes (ICD-9 codes 427.31; ICD-10 codes I48) from outpatient, inpatient and emergency room visits ascertained by CLG within 10 years of index date. The endpoint of the follow-up period for each participant was whichever of the following options occurred first: 1) date of the first AF event, 2) date of death, 3) date of last hospital visit, or 4) December 31st, 2017 (censored). Competing risk by all-cause mortality, obtained from both social security records and house staff notes, was considered to assess our primary endpoint.

### Statistical analysis

Baseline characteristics were summarized using median (Interquartile range, IQR) for continuous variables and percentage for categorical or nominal variables. We compared descriptive statistics by subgroups based on incidence or AF using Kruskal-Wallis test for continuous and chi-square test for categorical variables.

To counteract the competing risk of death, cumulative subhazard ratios of AF were estimated by competing-risk regression using Fine and Gray model adjusting for demographic (age, gender, race) and clinical (comorbidities) variables. Predicting factors of AF that were statistically significant (*p* <  0.05) from this multivariate competing risk regression were included in the risk score.

We followed methods previously described to develop our risk score [20, 21]. In summary, a continuous predictive score was constructed by attributing gradual values to such variables based on their beta coefficient. Next, we calculated a score for all patients from our cohort by calculating a point total using the risk score. The discrimination for both the multivariate competing-risk regression and our risk score was assessed using Wolber’s concordance index for prognostic models with competing risks [22]. Calibration was assessed by comparing observed and predicted number of AF events in quintiles of predicted risk. Finally, we conducted internal validation of our score by generating 1000 bootstrap samples with replacement. Analyses were performed using Stata 13 (StataCorp, College Station, Texas).

## Results

### Patients characteristics

A total of 9591 patients with grade 1 diastolic dysfunction were included in the study. The mean age for the study population was 66 ± 12 years, and 41.0% were male. The studied group was racially and ethnically diverse (Table 1): 13.3% were non-Hispanic White (NHW), 35.4% were non-Hispanic Black (NHB), 35.3% were Hispanic and 17.1% were Others (Asian, native Americans, Pacific Islanders, and multi-racial). There was a high percentage of PDD patients with hypertension (75.5%). In addition, high prevalences of obesity (38.7%) and diabetes (35.6%) were observed in this PDD population.

**Table 1:** Baseline characteristics of PDD patients

		AF – free (*N* = 9136)	Incident AF (*N* = 455)	*P*-value
Age (years), mean ± SD	66 ± 12	66 ± 12	70 ± 11	< 0.001
Male, n (%)	3935 (41.0)	3714 (40.7)	221 (48.6)	0.001
Mortality, n (%)	996 (10.4)	893 (9.8)	103 (22.6)	< 0.001
Race/Ethnicity, n (%)				< 0.001
White	1278 (13.3)	1176 (12.9)	102 (22.4)	
Black	3393 (35.4)	3232 (35.4)	161 (35.4)	
Hispanics	3384 (34.2)	3146 (34.4)	138 (30.3)	
Others	1636 (17.1)	1582 (17.3)	54 (11.9)	
SES, mean ± SD	−3.15 ± 2.8	−3.15 2.8	−3.01 ± 2.9	0.468
BMI (kg/m^2^), median (IQR)	28.3 (24.7–32.6)	28.3 (24.7–32.5)	28.3 (24.3–32.6)	0.856*
Comorbidities, n (%)				
HTN	7277 (75.9)	6895 (75.5)	382 (84.0)	< 0.001
MI	822 (8.6)	774 (8.5)	48 (10.6)	0.122
PAD	1250 (13.0)	1147 (12.6)	103 (22.6)	< 0.001
Stroke	1833 (19.6)	1770 (19.4)	113 (24.8)	< 0.001
DM	3416 (35.6)	3222 (35.3)	306 (42.6)	0.001
COPD	2519 (26.3)	2385 (26.1)	134 (29.4)	0.114
CKD	1798 (18.8)	1686 (18.5)	112 (24.6)	0.001
Malignancy	1428 (14.9)	1341 (14.7)	87 (19.1)	< 0.001
Obesity	3077 (38.7)	2933 (38.7)	144 (38.7)	1.000
Medications, n (%)				
ACEI/ARB	2324 (24.2)	2203 (24.1)	121 (26.6)	0.228
Beta-Blocker	1796 (18.7)	1699 (18.6)	97 (21.3)	0.146
Calcium Channel Blocker	1922 (20.0)	1828 (20.0)	94 (20.7)	0.735
Diuretics	2098 (21.9)	2001 (21.9)	97 (21.3)	0.769
Lifestyle risk factors, n (%)				
Alcohol use disorder	485 (5.1)	467 (5.1)	18 (4.0)	0.272
Active smoking	2267 (23.6)	2165 (23.7)	102 (22.4)	0.531

*SES* socioeconomic score, *BMI* body mass index, *IQR* interquartile range, *HTN* hypertension, *MI* myocardial infarction, *PAD* peripheral artery disease, *DM* diabetes mellitus, *COPD* chronic obstructive pulmonary disease, *CKD* chronic kidney disease, *ACEI/ARB* angiotensin-converting-enzyme inhibitor/angiotensin receptor blocker

^*^ *Medians are reported and compared using Kruskal-Wallis test*

During 10 years of follow-up, a total of 455 incident AF events (234 women and 221 men) occurred. A total of 996 (10.3%) people died during follow up, with a higher mortality in the patient group that developed new AF (22.6% vs. 9.8%, *p* <  0.001). Those who developed AF were older, more likely to be men, and had higher prevalence of baseline comorbidities including hypertension, diabetes, peripheral artery disease (PAD), stroke, chronic kidney disease and malignancy (Table 1). Additionally, there was a significantly higher percentage of non-Hispanic Whites in the patient group that developed AF during follow up than the group that remained AF-free (Tables 1, 22.4% vs. 12.9%, *p* <  0.001). The percentages of people with obesity were similar in between the groups. The proportion of alcohol use disorder and tobacco use disorder were 5 and 23.6%, respectively, with no significant differences between the two groups with and without incident AF. There was no difference in the use of individual anti-hypertensive medications between the group with incident AF and the rest of the cohort.

Echocardiographic variables were shown in Additional file 1: Table S1. Median left ventricular EF was 65% (IQR 60–68%) in total population; it was slightly lower in the individuals with incident AF than the others (EF 58% vs. 65%, *P* = 0.002) (Additional file 1: Table S1). Furthermore, PDD patients with incident AF presented with higher average left ventricular wall thickness and left ventricular volume (Additional file 1: Table S1). They also had significantly higher average left ventricular mass index (80.9 g/m^2^ vs. 75.8 g/m^2^, *p* = 0.002) and left atrial diameter index (19.3 mm/m^2^ vs. 18.8 mm/m^2^, *p* = 0.012) than the other PDD patients (Additional file 1: Table S1).

### Predicting factors of incidental AF in PDD patients

Multivariate competing-risk regression models were used to evaluate predicting factors for future AF in PDD patients. After adjusting for covariates, age (Subhazard Ratio (SHR) 1.02 per year, 95% CI 1.01–1.03, *p* <  0.001), male (SHR 1.34, 95% CI 1.09–1.63, *p* = 0.005), hypertension (SHR 1.52, 95% CI 1.13–2.05, p = 0.005), diabetes (SHR 1.34, 95% CI 1.09–1.66, *p* = 0.006) and PAD (SHR 1.70, 95% CI 1.33–2.17, p <  0.001) were found to independently associate with incident AF in the studied PDD cohort (Table 2). Non-Hispanic Blacks (SHR 0.67, 95% CI 0.50–0.90, *p* = 0.007), Hispanics (SHR 0.60, 95% CI 0.45–0.81, *p* = 0.001) and other race group (SHR 0.52, 95% CI 0.36–0.75, *p* <  0.001) independently associated with significantly lower risks of AF comparing to Non-Hispanic Whites in patients with PDD. Previous history of myocardial infarction, stroke, and chronic kidney disease were not independently associated with incident AF (Table 2). Finally, we also attempted to include echocardiogram data (LAD index and LV mass index) as covariates in the multivariate model; however they did not portend future AF events (Additional file 1: Table S2).

**Table 2:** Multivariate analysis showing predictors of Atrial Fibrillation in the PDD cohort

	SHR (95% CI)	*P*-values
Age	*1.02 (1.01–1.03)*	*< 0.001*
Male	*1.34 (1.09–1.63)*	*0.005*
Race (Ref = White)		
Black	*0.67 (0.50–0.90)*	*0.007*
Hispanics	*0.60 (0.45–0.81)*	*0.001*
Others	*0.52 (0.36–0.75)*	*< 0.001*
Co-morbidities		
HTN	*1.52 (1.13–2.05)*	*0.005*
DM	*1.34 (1.09–1.66)*	*0.006*
MI	1.03 (0.75–1.43)	0.17
PAD	*1.70 (1.33–2.17)*	*< 0.001*
Stroke	1.10 (0.88–1.39)	0.40
CKD	1.08 (0.84–1.39)	0.55
COPD	1.11 (0.89–1.37)	0.36
Malignancy	1.05 (0.81–1.36)	0.71

*HTN* hypertension, *DM* diabetes mellitus, *MI* myocardial infarction, *PAD* peripheral artery disease, *CKD* chronic kidney disease, *COPD* chronic obstructive pulmonary disease

### A risk score model to predict AF in PDD patients

Risk factors independently associated with incident AF in the multivariate analysis model were used to construct our risk score. Table 3 listed the predictors included in the risk score model; each of the variables was assigned a score proportional to its β-coefficient (shown in Additional file 1: Table S3). In order to facilitate memorization, an acronym SHARP-D (S, Sex; H, Hypertension; A, Age; R, Race; P, peripheral artery disease; D, diabetes) was created.

**Table 3:** Calculation of the SHARP-D Score for AF prediction in PDD cohort

Variables	Scores	
*Sex*	Male	1
	Female	0
*Hypertension*	No	0
	Yes	2
*Age (years)*	20 to < 30	−2
	30 to < 40	−1
	40 to < 50	0
	50 to < 60	1
	60 to < 70	2
	70 to < 80	3
	80 to < 90	4
	≥90	5
*Race*	White	0
	Black	−2
	Hispanics	−2
	Others	−3
*PAD*	No	0
	Yes	2
*Diabetes*	No	0
	Yes	1

**Fig. 1:**
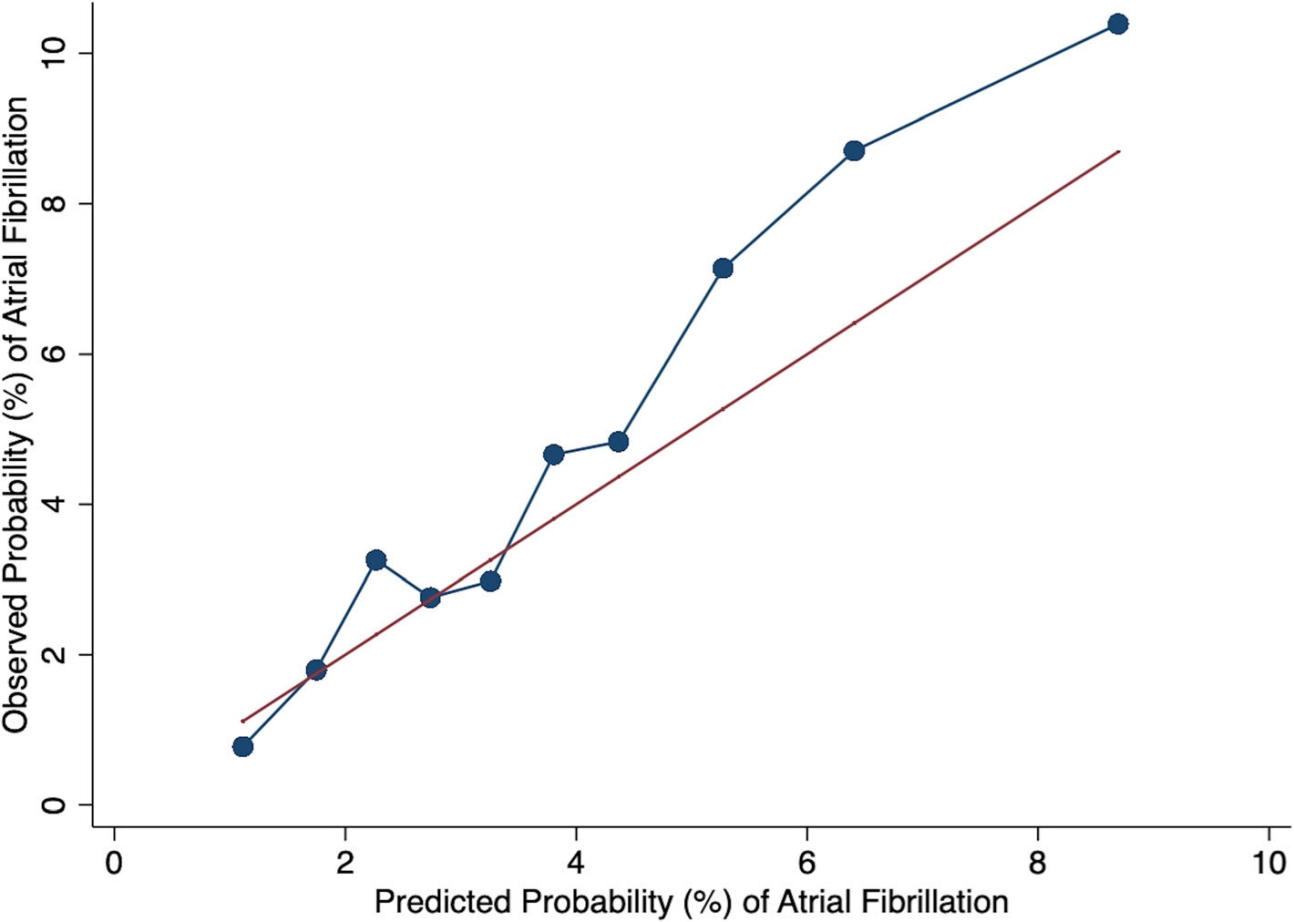
Calibration of risk prediction model. Correlation of observed and expected AF events by 10 deciles of the risk score was shown

The mean of SHARP-D score in the overall population was 2.8 ± 2.2 points (range − 4 to 11). The Wolber’s concordance index was 0.65 (0.63–0.68, *p* <  0.001) for the score, suggestive of good discrimination [22, 23]. The calibration of the model was assessed graphically by comparing the predicted probability of AF to the observed probability of AF at the end of follow-up across 10 deciles of predicted risk (Fig. 1). Overall, the SHARP-D score displayed good calibration with a modest degree of under-prediction in patients in the last three deciles of predicted risk (Fig. 1). Finally, for the internal validation of the SHARP-D score, we used 1000 bootstrap samples with replacement, and found a Wolber’s concordance index of 0.72 (95% CI 0.65–0.78), indicating that our score would perform well in subjects from populations similar to our cohort.

**Fig. 2:**
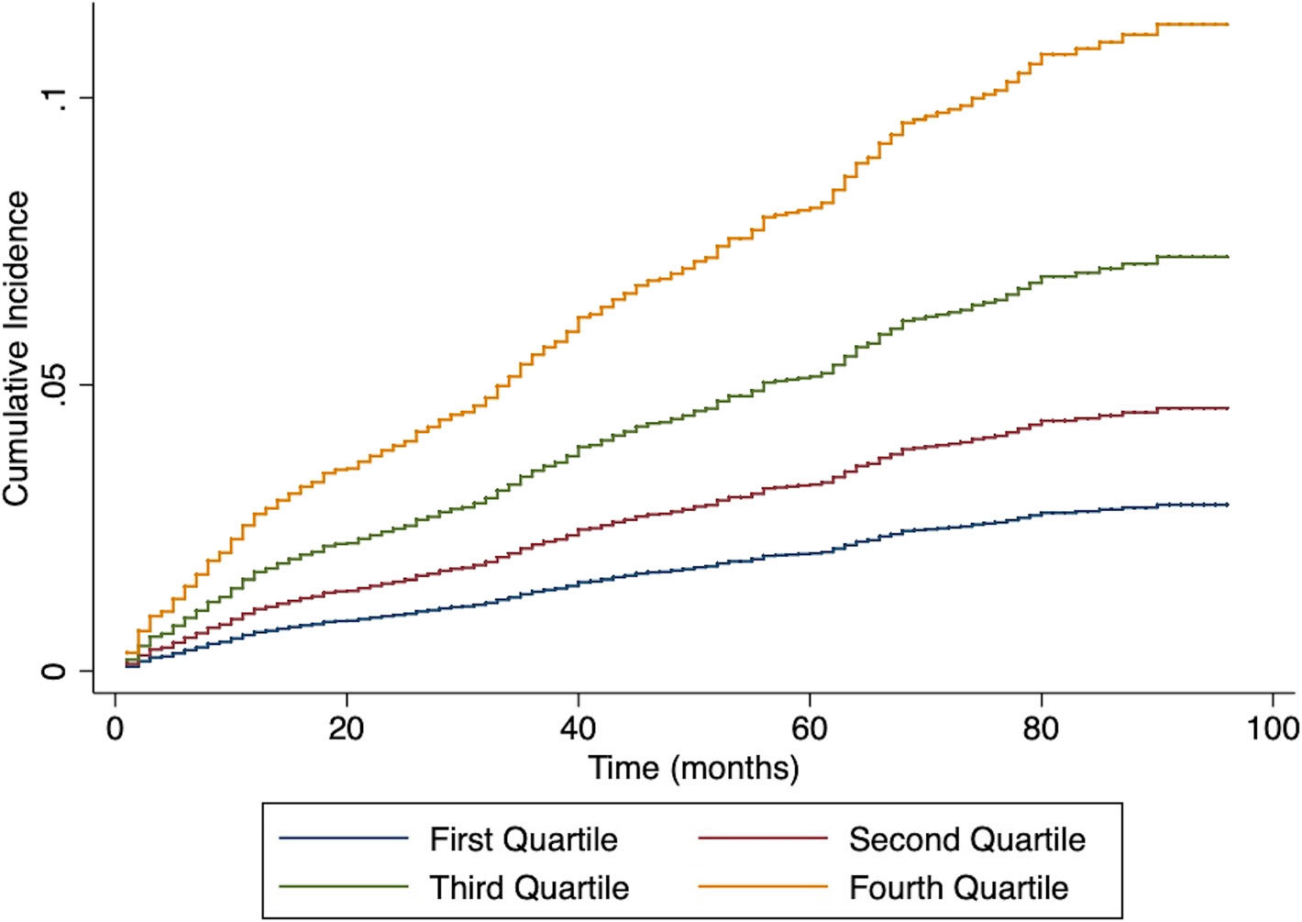
Observed AF events by quartiles of the SHARP-D score in PDD cohort

The study population was divided in quartiles using the calculated SHARP-D score, which corresponded with a predicted risk of incident AF in an individual patient **(**Additional file 1: Table S4). The cumulative incidences of AF in the four risk strata were shown in Fig. 2. Stratification on the four risk strata defined by quartiles of score points allowed us to effectively stratify patient at risk, and showed a strong gradation in the incidence of AF across four strata.

## Discussion

Left ventricular diastolic dysfunction has been shown to associate with increased risk of AF [10–14]. However, to the best of our knowledge, no studies in literature have identified predicting factors of incident AF or established predicting score models for assessing AF risk in PDD population. In this large institution-based study including 9591 patients with preclinical diastolic dysfunction of racial/ethnic diversity, we were able to show a set of independent predicting factors using a competing-risk approach that were associated with incident AF in PDD population. Furthermore, we constructed a simple risk scoring system with good discrimination that incorporates clinical variables, which makes the score easier to be implemented in clinical practice for AF preventive measures and early diagnosis.

PDD is a common phenomenon in the general population, with a prevalence of 27–36% [5–7]. The majority of individuals with PDD have grade 1 diastolic dysfunction, of which prevalence is reported 20.6–23.5% [5, 24]. Hence we selected patients with grade 1 diastolic dysfunction to represent PDD. Higher severity of diastolic dysfunction often accompanies symptoms of diastolic heart failure, which might not be diagnosed clinically in a prompt fashion. Often considered as a mild pathology and neglected in clinical practice, PDD is associated with increased risk of heart failure and overall mortality [5, 24]. Diastolic dysfunction is also known to associate with an increased risk of incident AF [11, 12, 14], highlighting the clinical significance of preventive measures in this population.

In comparison to the Framingham AF risk prediction study, which identified age, sex, body-mass index, systolic blood pressure, treatment for hypertension, PR interval, murmurs and heart failure as AF predictors in general population [25], we showed that age, male sex, White race, chronic comorbidities including hypertension, diabetes, and PAD are independently associated with future AF in PDD individuals. Distinctively, we showed that diabetes (HR 1.34, 95% CI 1.09–1.66) and PAD (HR 1.70, 95% CI 1.33–2.17) are significant predictors of incident AF. This difference could be partially attributed to patient population (general population vs. patients with PDD, as well as racial and ethnicity differences).

Additionally, the Framingham AF risk score for general population is deprived from a White-predominant population [25], who are known to carry a higher risk of AF than people of other racial groups [26, 27]. The Framingham AF risk model has been shown to operate better in Whites than Blacks [28]. Our racially diverse population enabled us to include race (White, Black, Hispanic, and Others) as an important factor to assist in AF risk stratification among PDD individuals.

We constructed a clinical risk score (SHARP-D) to assist predict AF risk in individuals with PDD. Since the SHARP-D score showed better calibration in the first five deciles of predicted risk, it is reasonable to suggest that lower actual risk would be better predicted by our score compared to higher actual risk. Despite the likely underestimation of the incident AF risk in patients with higher risk profiles, our scoring system could fulfill the task of risk stratification and identification of high-risk individuals with PDD.

We propose two aspects of preventive measures for high-risk patients with PDD: management of risk factors and early diagnosis of subclinical AF. Although there is lack of direct evidence that tight control of hypertension or diabetes reduces AF risk, studies have shown the systolic blood pressure as an independent risk factor of incident AF in the Framingham study [25]. Further, the level of hemoglobin A1c – a marker of glycemic control in diabetic patients, correlates with the risk of incident AF in a large cohort Japanese study [29]. Additionally, aggressive control of risk factors including hypertension, diabetes has been shown to reduce long-term success in maintaining sinus rhythm after AF ablation [30]. Therefore, further optimization of cardiovascular risk factors including hypertension and diabetes will still likely be beneficial in reducing AF risk in high-risk patients. On the other hand, it is unclear if there is a causal relationship between PAD and AF; rather, the association might be attributed to the shared cardiovascular risk factors [31].

Furthermore, using the SHARP-D score in individuals with PDD, close monitoring and early diagnosis of AF in the high-risk population could help preventing secondary complications of AF including stroke. *Marfella R* et al. showed a remarkably higher prevalence of subclinical AF episodes in diabetic patients compared with matched healthy individuals (11% vs. 1.6%, *p* <  0.0001) [32]. Detected by 48-h holter monitoring, these silent AF episodes were associated with significantly increased stroke risk (HR 4.6, 95% CI 2.7–9.1) [32]. Therefore, more frequent cardiac monitor in high-risk patient identified by the risk score could potentially increase the sensitivity of subclinical AF diagnosis.

The strengths of our study include large sample volume of a racially diverse population and application of competing risk model to assess the future AF without the competing effect of mortality. There are several limitations of our study. Firstly, the incidence of AF is based on medical records and likely underestimated given the lack of electrocardiogram data and underdiagnosis of paroxysmal AF by nature. Secondly, the 2016 ASE/EACVI algorithm to grade diastolic dysfunction requires more parameters such as tricuspid regurgitation peak velocity and left atrial volume index [16]. Due to insufficient availability of these parameters in earlier echocardiographic studies, and impractical nature to apply the complicated algorithm to a large-volume retrospective study, we used criteria of E/A ≤ 0.8 and E/e’ < 10 to define grade 1 diastolic function. Similar criteria have been used in literature to define grade 1 diastolic dysfunction but used E/A ration ≤0.75 and E/e’ < 10 [15, 33]. We applied criteria of E/A ratio ≤ 0.8 here instead of 0.75 in accordance with the 2016 ASE/EACVI guideline for defining grade 1 diastolic dysfunction. The change of this threshold did not significantly affect our studied cohort. Thirdly, we used LAD index rather than LA volume index as a parameter of left atrial size due to insufficient data on LA volume. Although LA volume index has been shown to associate more closely with cardiovascular outcome in general population [34], LAD index and LA volume index correlate well with each other [34, 35].

## Conclusions

In a large, multi-racial, hospital-based population with PDD, we identified a set of clinical predictors of AF that served to construct a simple risk scoring system that predicted events of AF reasonably well, and therefore, will help identify high-risk individuals in order to better direct early preventive measures.

## Additional file

Additional file 1: **Table S1** Echocardiography data. Baseline echocardiography data were shown in PDD patients with and without incident AF during follow up. **Table S2** Multivariate analysis showing predictors of AF in PDD patients including echocardiographic covariates. **Table S3** β – coefficients for factors included in the risk score. **Table S4** The quartiles of patients with PDD according to the SHARP-D score, and the predicted risk for incident AF accordingly. (DOCX 26 kb)

## Abbreviations

AF: Atrial fibrillation
BSA: Body surface area
CI: Confidence interval
LAD: Left atrial diameter
LV: Left ventricle
LVEF: Left ventricular ejection fraction
NHB: Non-Hispanic Black
NHW: Non-Hispanic White
PAD: Peripheral artery disease
PDD: Preclinical diastolic dysfunction
SES: Socioeconomic score
SHR: Subhazard ratio
