# Supplementary material for: A risk score for predicting atrial fibrillation in individuals with preclinical diastolic dysfunction: a retrospective study in a single large urban center in the United States
Source: BMC Cardiovasc Disord. 2019 Feb 27;19:47. doi: 10.1186/s12872-019-1024-4 (PMC6391831; doi:10.1186/s12872-019-1024-4)
Supplement: Supplementary file 1 — Table S1 Echocardiography data. Baseline echocardiography data were shown in PDD patients with and without incident AF during follow up. Table S2 Multivariate analysis showing predictors of AF in PDD patients including echocardiographic covariates. Table S3 β – coefficients for factors included in the risk score. Table S4 The quartiles of patients with PDD according to the SHARP-D score, and the predicted risk for incident AF accordingly. (DOCX 26 kb) [file 12872_2019_1024_MOESM1_ESM.docx]

**Table S1** Echocardiography data.

| **Median**  **(IQR)** | **Available data**  **(%)** | **Total**  **(N = 9591)** | **No AF**  **(N = 9136)** | **AF**  **(N = 455)** | **P-value** |
| --- | --- | --- | --- | --- | --- |
| **EF (%)** | 9592  (100%) | 65  (60-67.5) | 65  (60-68) | 58  (50-68) | 0.002 |
| **LAD (mm)** | 663  (93.1%) | 35  (31-38) | 35.0  (31.38) | 36  (32-40) | < 0.001 |
| **LAD index (mm/m^2^)** | 7416  (77.3%) | 18.9  (16.8-20.9) | 18,8  (16.8-20.9) | 19.3  (17.0-21.6) | 0.012 |
| **IVSd (mm)** | 9193  (95.9%) | 10  (9-11) | 10  (9-11) | 10  (9-12) | 0.007 |
| **LVPWd (mm)** | 9192  (95.8%) | 9  (8-11) | 9  (8-11) | 10  (8-11) | 0.038 |
| **LVEDD (mm)** | 9213  (96.1%) | 44  (40-48) | 44  (40-48) | 45  (41-49) | 0.009 |
| **LV Mass (g)** | 9179  (95.7%) | 138  (114-170) | 138  (114-170) | 152  (118-185) | < 0.001 |
| **LV Mass index (g/m^2^)** | 7641  (79.7%) | 76.0  (63.2-90.9) | 75.8  (63.2-90.6) | 80.9  (64.7-95.9) | 0.002 |

IQR, interquartile range; EF, ejection fraction; LAD, left atrial diameter; LAD index, LAD adjusted by body surface area; LVEDD, left ventricular end-diastolic diameter; IVSd, intraventricular septal thickness at end-diastole; LVPWd, left ventricular posterior wall thickness at end-diastole; LV mass, left ventricular mass; LV mass index, LV mass adjusted by body surface area.

**Table S2** Multivariate analysis in predicting AF in PDD patients (including echocardiographic covariates).

|  | **SHR (95% CI)** | **P-values** |
| --- | --- | --- |
| **Age** | *1.02 (1.01-1.03)* | *0.001* |
| **Male** | *1.32 (1.04-1.68)* | *0.022* |
| **Race (Ref = White)** |  |  |
| **Black** | *0.65 (0.47-0.91)* | *0.001* |
| **Hispanics** | *0.57 (0.41-0.80)* | *0.001* |
| **Others** | *0.56 (0.37-0.85)* | *0.006* |
| **Co-morbidities** |  |  |
| **HTN** | *1.47 (1.04-2.06)* | *0.028* |
| **DM** | *1.27 (1.00-1.62)* | *0.05* |
| **MI** | 1.12 (0.79-1.59) | 0.54 |
| **PAD** | *1.88 (1.43-2.48)* | *< 0.001* |
| **Stroke** | 1.06 (0.81-1.39) | 0.66 |
| **CKD** | 1.12 (0.84-1.49) | 0.42 |
| **COPD** | 1.13 (0.881.49) | 0.42 |
| **Malignancy** | 1.05 (0.78-1.42) | 0.73 |
| **Echocardiogram** |  |  |
| **LAD index** | 1.02 (0.98-1.07) | 0.267 |
| **LV mass index** | 1.01 (1.00-1.01) | 0.05 |

HTN, hypertension; DM, diabetes mellitus; MI, myocardial infarction; PAD, peripheral artery disease; CKD, chronic kidney disease; COPD, chronic obstructive pulmonary disease; LAD, left atrial diameter; LAD index, LAD adjusted by body surface area; LV mass index, left ventricular mass adjusted by body surface area.

**Table S3** β – coefficients for factors included in the risk score.

| **Variables** | **Scores** | β**-coefficient** |
| --- | --- | --- |
| ***S****ex* | Male  Female | REF  0.28 |
| ***H****ypertension* | No | REF |
|  | Yes | 0.42 |
| ***A****ge (years)* | 20 to <30 | REF |
|  | 30 to <40 | 11.9 |
|  | 40 to <50 | 11.5 |
|  | 50 to <60 | 11.5 |
|  | 60 to <70 | 12.0 |
|  | 70 to <80 | 12.1 |
|  | 80 to <90 | 12.1 |
|  | ≥90 | 12.3 |
| ***R****ace* | White | REF |
|  | Black | -0.42 |
|  | Hispanics | -0.54 |
|  | Others | -0.67 |
| ***P****AD* | No | REF |
|  | Yes | 0.54 |
| ***D****iabetes* | No | REF |
|  | Yes | 0.29 |

**Table S4** The quartiles of SHARP-D scores and the predicted AF risk.

| **Quartiles** | **Score Range** | **Predicted Risk (%)** |
| --- | --- | --- |
| **First** | *-4 to 1* | *1.9* |
| **Second** | *2 to 3* | *3.4* |
| **Third** | *4* | *4.7* |
| **Fourth** | *5 to 11* | *6.6* |
